# Stem cells: a window of opportunity in low-dimensional EMT space

**DOI:** 10.18632/oncotarget.25852

**Published:** 2018-08-07

**Authors:** Qing Nie

**Affiliations:** Qing Nie: Department of Developmental and Cell Biology, and Department of Mathematics, University of California, Irvine, CA, USA

**Keywords:** intermediate cell state, EMT, stem cells, modeling, heterogeneity

Cells often need to dynamically adapt their fates to perform different biological functions. Sometimes cells must possess stemness in order to produce multiple lineages of cells while at the same time these cells may need to migrate to new spatial locations. One prime example is the epithelial-mesenchymal transition (EMT), during which epithelial cells modify their adhesion properties and gain the capacity to migrate; In the meantime lineage-specific stem/progenitor cells drive tissue growth whereas progeny of stem cells sometimes revert back to stem cell states for their functional flexibility [1]. How do cells handle and coordinate such multiple challenging tasks simultaneously?

The answer appears to lie in an intermediate cell state (ICS). Through mathematical modeling and computational analysis of the EMT process and cancer stem cells (CSCs), Bocci et al. scrutinized interconnections among different cellular states (or fates) controlled by regulatory circuits of EMT and CSCs [2]. A previously identified ICS, which exhibits hybrid (or partial) gene expression profiles of both epithelial and mesenchymal states, was first found to intimately connect with stemness of the cells during the EMT process. The ICS are located at the “middle” of a low-dimensional gene expression space when dimension reduction is carried out using measurement of a one-dimensional EMT score.

**Figure 1:**
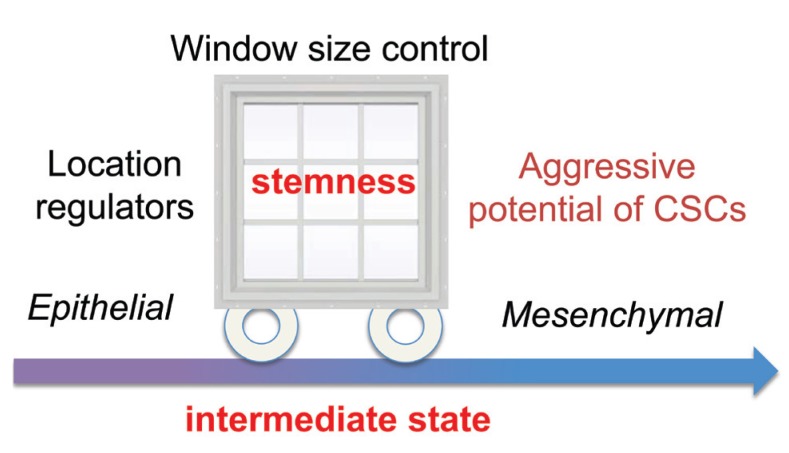
A window of opportunity for stemness in an EMT space

Interestingly, the location of the ICS in the low-dimensional gene expression space can shift towards either end (i.e. E or M) of the EMT spectrum, creating a window of opportunity for stemness (Figure 1). Different locations of the window can enable production of stem cells with different migration potential necessary for various epithelial tissues. Crosstalk between EMT genes and stemness genes was found to regulate the location of the window, allowing either stem-like epithelial cells or stem-like mesenchymal cells.

Another regulator of this stemness window is Notch signaling; a cell-cell contact mechanism that might be critical to collective migration as well as serving as a communication mechanism to relay stemness fate information from one cell to its neighbors. Intermediate levels of both Notch and Jagged expression are associated with the ICS that exhibits stemness. Through feedback with the EMT process [3], Notch signaling thus couples and “matches” the ICS with the required gene expression profiles of stemness.

The size of the window is controlled by three input signals: NF-κB signaling level, EMT induction, or Notch activation. For example, a strong EMT-induction shrinks the window while a NF-κB overexpression can enlarge the window. A small window implies limited opportunities for stemness, leading to the entire EMT space containing only epithelial cells or/and mesenchymal cells. With different combinations of the three signals, various possible combinations of different types of cells can exist, however, stem-like epithelial cells and stem-like mesenchymal cells can’t co-exist. If both types of stem-like cells are needed in the same tissue, two or more intermediate states in the EMT space may be important [4].

Investigations of expression data of CSCs from several cancer subtypes recapitulate various profiles of stemness, EMT, and Notch signaling genes that are predicted by modeling. By analyzing location and size of the stemness for cancer cells in lung, breast, thyroid, and glioblastoma, the authors suggest each cancer type possesses very different migration potential and stemness capability. If one could reduce size of the window or shift the window away from the mesenchymal end of the EMT space, the tumor would be less aggressive in growth or invasiveness (Figure 1). By modeling the effect of metformin, an anti-diabetic drug that was recently observed to have an anticancer effect, the authors were able to recapitulate several experimental observations, and to predict specific molecular mechanisms on shifting the window or/and reducing its size.

This work has provided a framework to tie together molecular mechanisms for otherwise unrelated - and often confusing - biological observations regarding EMT, stem-like traits and Notch signaling [2]. It further elucidates the importance of intermediate cell states and the transitions between them [5]. The new insights learned from this integrated modeling and data analysis provide us with a better understanding of how molecular mechanisms coordinate the migration and stemness of cells, and the window of opportunity model naturally explains the considerable cellular heterogeneity observed in many cancers.
